# Daily intake of antioxidants in relation to survival among adult patients diagnosed with malignant glioma

**DOI:** 10.1186/1471-2407-10-215

**Published:** 2010-05-19

**Authors:** Gerald N DeLorenze, Lucie McCoy, Ai-Lin Tsai, Charles P Quesenberry, Terri Rice, Dora Il'yasova, Margaret Wrensch

**Affiliations:** 1 Division of Research, Kaiser Permanente Northern California, 2000 Broadway, Oakland, CA, USA; 2 Department of Neurological Surgery, University of California San Francisco, 44 Page St, San Francisco, USA; 3 Department of Community and Family Medicine, Duke University Medical Center, 2424 Erwin Rd, Durham, NC, USA

## Abstract

**Background:**

Malignant glioma is a rare cancer with poor survival. The influence of diet and antioxidant intake on glioma survival is not well understood. The current study examines the association between antioxidant intake and survival after glioma diagnosis.

**Methods:**

Adult patients diagnosed with malignant glioma during 1991-1994 and 1997-2001 were enrolled in a population-based study. Diagnosis was confirmed by review of pathology specimens. A modified food-frequency questionnaire interview was completed by each glioma patient or a designated proxy. Intake of each food item was converted to grams consumed/day. From this nutrient database, 16 antioxidants, calcium, a total antioxidant index and 3 macronutrients were available for survival analysis. Cox regression estimated mortality hazard ratios associated with each nutrient and the antioxidant index adjusting for potential confounders. Nutrient values were categorized into tertiles. Models were stratified by histology (Grades II, III, and IV) and conducted for all (including proxy) subjects and for a subset of self-reported subjects.

**Results:**

Geometric mean values for 11 fat-soluble and 6 water-soluble individual antioxidants, antioxidant index and 3 macronutrients were virtually the same when comparing all cases (n = 748) to self-reported cases only (n = 450). For patients diagnosed with Grade II and Grade III histology, moderate (915.8-2118.3 mcg) intake of fat-soluble lycopene was associated with poorer survival when compared to low intake (0.0-914.8 mcg), for self-reported cases only. High intake of vitamin E and moderate/high intake of secoisolariciresinol among Grade III patients indicated greater survival for all cases. In Grade IV patients, moderate/high intake of cryptoxanthin and high intake of secoisolariciresinol were associated with poorer survival among all cases. Among Grade II patients, moderate intake of water-soluble folate was associated with greater survival for all cases; high intake of vitamin C and genistein and the highest level of the antioxidant index were associated with poorer survival for all cases.

**Conclusions:**

The associations observed in our study suggest that the influence of some antioxidants on survival following a diagnosis of malignant glioma are inconsistent and vary by histology group. Further research in a large sample of glioma patients is needed to confirm/refute our results.

## Background

Mortality following diagnosis of malignant brain tumors varies by histologic type and other risk factors. Between 1991 and 2000, adults diagnosed with malignant glioma had median survival times that ranged from 7 months for highest grade tumors (gliomablastoma multiforme) to 91 months for lower grade tumors (oligodendroglioma) [1]. The majority of glioma patients are diagnosed with gliomablastoma multiforme (GBM), and 5-year survivorship is < 3%. Among patient characteristics that are associated with differential survival rates following a glioma diagnosis, only age appears to be as strong a predictor as histology [2]. Other risk factors such as tumor location and the magnitude of tumor resection [3-6], molecular and genetic markers [7-13], and Karnofsky performanace status [14-20] have been examined in a number of studies of survival in glioma patients. However, the potential association of diet, in general, and antioxidant intake, in particular, with survival after glioma diagnosis in human populations has been only sparsely investigated [21,22].

Oxygen-derived free radicals produced by lipid peroxidation are believed to play an important role in cancer development [23,24]. Antioxidants can act as reducing agents to prevent oxidative reactions by either scavenging reactive oxygen species (ROS) or inhibiting cellular signaling enzymes such as protein kinase C (PKC) [25,26]. Proliferation of malignant gliomas can be induced by PKC isoforms through several signaling pathways [27-30]. Endogenous antioxidant enzymes are one of the brain's natural defense mechanisms to remove the highly toxic hydrogen peroxide molecule; decreased activity of glutathione peroxidase and reductase have been found in glioblastoma tissue [31-33]. Dietary antioxidants, including isoflavones and polyphenols, have been shown in laboratory studies to enhance growth restriction of cancer cells in general [34] and glioma cells in particular [35,36]. However other studies have indicated that high concentrations of non-enzymatic antioxidants, including carotenoids and flavonoids, can exhibit pro-oxidant behavior, particularly in the presence of redox-active metals (copper, iron) and a high pH [26,37]. There is also evidence that the combination of cigarette smoking and antioxidant intake can diminish the effects of radiotherapy for certain cancers [38].

With respect to a potential effect of antioxidants on survival of patients with gliomas, two opposing hypotheses can be formulated. First, because brain tissues are one of the most oxidative environments and therefore susceptible to radiation damage, antioxidants may protect normal brain tissues from radiation damage and lead to better survival [39]. On the other hand, antioxidants may make the glioma more resistant to tumor killing by radiation [40], possibly leading to poorer patient survival. Given this lack of consensus it is important then to examine the potential association between antioxidant intake and patient survival in a large population-based cohort of adults diagnosed with malignant glioma.

In the current study, we examine patterns of survival after a diagnosis of malignant glioma in relation to intake of dietary antioxidants and other nutrients. We estimate the contribution of antioxidants and other nurtients, both separately and in combination with each other, to glioma survival adjusting for known risk factors and confounders. Our results are stratified by histology group and by type of interview, i.e. patient self-report or interview with the patient's designated proxy.

## Methods

### Study population

The ascertainment of glioma cases and study subject eligibility requirements have been described in detail by Wiemels et al. [41] and Wrensch et al. [42]. In summary, any adult (ages ≥ 20) with an incident diagnosis of glioma (ICD-O morphology codes 9380-9481) between August 1991 and April 1994 (series 1) and May 1997 and August 1999 (series 2) residing anywhere in the six county San Francisco-Oakland MRSA ould meet study eligibility criteria [1]. Potential glioma cases were identified using rapid case ascertainment (RCA) provided by a Surveillance Epidemiology and End Results (SEER) participating registry, the Northern California Cancer Center (NCCC). Subjects or their designated proxies provided written consent to obtain and review pathology specimens and records to confirm a glioma diagnosis. Consenting patients or their proxies completed surveys concerning a number of characteristics and potential risk factors. Other glioma cases were not included as study subjects because either consent was not obtained, neuropathology review was not possible or revealed a diagnosis not eligible for the study.

### Neuropathology Review

Pathology records and specimens, provided by the diagnosing hospitals, were reviewed by a neuropathologist [43]. WHO criteria [44] were used to classify all tumors. GBM corresponds to WHO grade IV, anaplastic astrocytoma (AA) to WHO grade III, and astrocytoma to WHO grade II.

### Determination of Vital Status

NCCC-SEER, which routinely ascertains vital status via California State mortality tapes and the National Death Index, provided vital status for the study subjects in December 2002 [1]. For cases not known to be deceased, a letter was sent to the last known address, followed by a telephone call. Mortality was updated from SEER in December, 2008. For a small number of cases who could not be located, date of last contact (as determined by NCCC-SEER) was used. Patients not known to be dead were censored at date of last contact.

### Dietary questionnaires and interviews

#### Series I

Eligible cases or designated proxies were given an in-person interview at the subjects' home or other location of their choice [45]. Subjects received a dietary questionnaire in advance, which was collected or completed at the in-person interview. The temporal period for all questions was the year preceding the glioma diagnosis.

A detailed description of the Series I questionnaire has been provided in Tedeschi-Blok et al [46]. Briefly, a 79-item food-frequency questionnaire was modified from the US National Cancer Institutes' (Block's) Health Habits and History Questionnaire [47]and the Los Angeles Glioma and Meningioma Study Questionnaire [48]. Subjects reported their usual frequencies of consumption of each food item. The frequency choices included: 'never', 'less than 1 per month', '1 per month', '2-3 per month', '1 per week', '2 per week', '3-4 per week', '5-6 per week', '1 per day', and '2 or more per day'. Portion sizes were not asked, but were estimated using gender- and age- specific values from the Block and colleagues' National Cancer Institute's Health Habits and History Questionnaire [47].

#### Series II

Series II eligible cases or designated proxies were approached in a manner similar to Series I. However instead of receiving a self-administered diet questionnaire in advance, interviewers asked the diet questions in Series II. The 96-item Series II diet questionnaire was modified from the US National Cancer Institutes' (Block's) Health Habits and History Questionnaire. Modifications [47]. The frequency choices included: 'never or less than 1 per month', '1 per month', '2-3 per month, '1 per week', '2 per week', '3-4 per week', '5-6 per week', '1 per day'.

#### Series I & II

For eligible subjects who did not wish to participate in the full interview, a 5-minute phone interview was administered using an abbreviated questionnaire asking basic demographic and dietary questions. This permitted evaluation of major differences in dietary consumption between those cases who participated in the full interview and those who did not.

### Dietary analyses

Assignment of reported food frequency choices into levels of specific nutrients has been previously described in detail by Tedeschi-Blok et al [46]. Intake of each food item was converted to grams consumed per day by applying an appropriate algorithm for each series. For both series, a nutrient database included a total of 27 nutrients per 100 grams for each food item. Of the 27 nutrients, 16 antioxidants plus calcium and 3 macronutrients were available for our survival analysis. A total daily antioxidant index intake was calculated by summing the product of grams consumed over all food items and units of antioxidant index per gram from an antioxidant index database for fruit, vegetable, juice and tea food items [49-51]. Antioxidant index values were units of micromoles Trolox equivalents per gram of food. Trolox equivalents per gram of food were measured by the oxygen radical absorbance capacity (ORAC) assay [50,51].

### Use of Alcohol and Tobacco

The Series I and II surveys included questions concerning alcohol consumption (age at first drink; weekly consumption of wine, beer and liquor) and cigarette smoking (age began smoking; number of cigarettes per day; number of years of active smoking). Because both alcohol and cigarette smoke are oxidants and have been shown to be related to oxidative stress in the brain and the expression of antioxidants (e.g., vitamin C and beta-carotene) [52,53], responses to these questions were included in the study analyses.

### Determination of Treatment Information

Treatment information and other clinical characteristics was obtained from NCCC-SEER, medical record abstraction, and a clinical trials database from UCSF. NCCC-SEER treatment information includes summary data for surgery, radiation, and chemotherapy. NCCC-SEER provided a summary of curative treatment information given as the first course of therapy following diagnosis. Information from medical record abstraction included Karnofsky performance status (KPS) at time of diagnosis, tumor location, surgical resection, radiation, and chemotherapy. The UCSF Neuro-Oncology Service database contained these data items for patients entered in clinical trials. Since KPS was not available for many subjects, it was not included in these analyses.

### Statistical Analysis

Preliminary data analysis included an examination of the univariate distributions of nutrients and potential confounders including patient demographics (age, gender, race, education, income, marital status), histology group, treatment, series, comorbid conditions (epilepsy, chicken pox, shingles, allergies), Karnofsky score, alcohol and tobacco use for all glioma cases (including interviews completed by a designated proxy) and for only cases who were interviewed directly (no proxy). Several of these potential confounders, age at diagnosis in particular, have been previously reported as significantly associated with mortality following a glioma diagnosis in a survival analysis of the same patients included in the current study [1]. Descriptive statistics included geometric mean nutrient intake (given the skewness in nutrient distributions) adjusted for total calories. Adjusted geometric means were obtained via a linear regression of log nutrient on calories and calculating the anti-log of the least squares mean. Preliminary analyses also included an examination of each nutrient and potential confounder in relation to survival via Kaplan-Meier survival curves and log-rank tests.

Based upon the results of the univariate analyses, Cox proportional hazards models were used for point and interval estimation of mortality hazard ratios associated with each antioxidant, antioxidant index, and other nutrients adjusting for potential confounders. Regression analyses were stratified by grade and conducted using all (including proxy interviews) glioma cases and in the subset of self-reported cases. Primary analyses examined nutrients categorized in tertiles to allow examination of dose response and threshold effects. Given previous studies in this and other cohorts [1,3-6,15,54], age and treatment (radiation, chemotherapy, surgery: all as dichotomous yes/no) were a priori included in all regression analyses. As is standard in analyses of nutrients in relation to health outcomes, total caloric intake was also a priori included in all regression models. Given results from our preliminary analyses as well as evidence from previous studies of risk factors [38,41,45] associated with malignant glioma, education, marital status, alcohol (age at first alcoholic drink) and tobacco use (pack years of cigarette smoking) were included as covariates in the regression analysis. In addition, for any significant base-model adjusted nutrient survival associations that were observed, we did a check for other potential confounders (e.g. income, comorbid conditions, protein intake), although none were found to appreciably change hazard ratio (HR) estimates. All tests of significance in this analysis were 2-sided, and all statistical analyses were completed using SAS statistical software (Version 9.1, Cary, NC). Final models were selected based upon statistical significance.

## Results

The distribution of demographic, clinical and behaviorial characteristics is presented in Table 1, which is stratified for all cases (including interviews completed by proxy) and for self-reported cases (case was interviewed) only. Self-reported (SR) cases were somewhat younger in age at the time of diagnosis when compared to all cases. The distribution of gender and race categories was similar in both groups. Self-reported cases were more likely to have completed at least one year of college, had a higher proportion of Grade II (histology) tumors, and a lower proportion of Grade IV tumors in comparison to all cases. Both groups showed similar distributions in the categories for age at first alcoholic drink and smoking (pack-years). For glioma-related treatment the self-reported cases had somewhat higher rates of receiving surgical resection, radiotherapy and chemotherapy when compared to all cases.

**Table 1:** Distribution of patient characteristics; San Francisco Bay Area Adult Glioma Study, 1991-2000

* Characteristics*	* All cases (N = 748)*	* Self-reported cases (N = 450)*
Age (median ± SE)	55.7 ± 0.6	49.8 ± 0.7
Gender (% male)	57.1%	59.1%
Ethnicity (% White)	83.4%	84.2%
Education		
< 12 years	12.2%	6.4%
High school graduate	24.2%	20.9%
≥ 1 year college	63.6%	72.7%
Histology Group		
Grade II^†^	19.1%	28.2%
Grade III^‡^	17.4%	19.3%
Grade IV*	63.5%	52.4%
Treatment		
Surgical Resection	83.0%	91.8%
Radiotherapy	73.7%	83.1%
Chemotherapy	20.3%	26.9%
Age at first drink		
Never drank alcohol	9.2%	8.0%
<18 years old	30.4%	31.3%
18 - 20 years old	33.2%	34.7%
≥21 years old	26.6%	26.0%
Unknown	0.7%	0%
Total Packyears		
Never smoked	43.5%	46.0%
> 0 to ≤ 18.5	25.4%	30.0%
> 18.5	25.9%	22.0%
Unknown	5.2%	2.0%

^† ^includes 44 astrocytomas, 63 oligodendrogliomas, 36 oligoastrocytomas.

^‡ ^includes 90 astrocytomas, 26 oligodendrogliomas, 14 oligoastrocytomas.

*all (n = 475) glioblastomas.

Geometric means of the total daily antioxidants and other nutrients consumed adjusted for total calories among all cases are shown in Table 2. The geometric mean values for the Antioxidant Index are virtually the same when comparing all cases to self-reported cases only. A similar pattern is observed for most of the 11 fat-soluble and 7 water-soluble individual antioxidants and the 3 macronutrients listed in Table 2.

**Table 2:** Antioxidant and nutrient geometric mean values (adjusted for total calorie intake)

Nutrient	All cases (N = 748)	Self-reported cases (N = 450)
Fat-soluble:		
Vitamin E (mg)	7.8	7.6
Carotenoids (mcg)	3441.1	3327.4
Alpha-carotene (mcg)	589.9	554.8
Beta-carotene (mcg)	2783.5	2696.4
Lutein (mcg)	1889.5	1906.2
Lycopene (mcg)	1230.0	1277.1
Cryptoxanthin (mcg)	104.6	110.2
Coumestrol (mcg)	107.7	111.3
Matairesinol (mcg)	23.4	23.6
Secoisolariciresinol (mcg)	105.9	105.4
Linoleic acid (g)	11.2	10.9
Water-soluble:		
Vitamin C (mg)	107.5	108.6
Formononetin (mcg)	14.3	15.2
Genistein (mcg)	230.8	248.3
Daidzein (mcg)	352.1	370.8
Biochanin A (mcg)	22.8	23.4
Calcium (mg)	642.6	626.3
Folate (mcg)	359.7	355.9
		
Antioxidant index	2442.9	2445.8
		
Macronutrients:		
Total Protein (g)	59.8	58.6
Total Fat (g)	58.3	56.0
Total Cholesterol (mg)	210.4	202.8
		
Total Kcal (unadjusted)	1634.0	1599.8

Hazard ratios for tertiles of daily intake of fat-soluble antioxidants adjusted for age at glioma diagnosis, education, marital status, treatment, cigarette smoking (pack-years), alcohol (age at first alcoholic drink), and total calories, stratified by proxy status (all cases vs self-reported cases only) and histology group (Grade II, III, and IV) are presented in Table 3. Examining these results for those that were statistically significant, we found that the hazard ratio for moderate intake of lycopene (HR = 2.31, 95% CI = 1.12 to 4.75, for second tertile compared to lowest tertile, in self-reported cases only) was elevated within the Grade II histology group. Similarly moderate intake of lycopene among patients in the Grade III histology group was associated with poorer survival. Hazard ratios for high intake of vitamin E (HR = 0.37, 95% CI = 0.17 to 0.81, highest tertile compared to lowest tertile, in all cases) and for both moderate and high intake of secoisolariciresinol (for second tertile and for highest tertile compared to lowest tertile, in all cases) among Grade III patients indicate enhanced survival. Moderate intake of alpha-carotene (HR = 2.12, 95% CI = 1.08 to 4.18, for second tertile compared to lowest tertile, in self-reported cases only) among Grade III patients was associated with poorer survival. In the Grade IV histology group, moderate to high intake of cryptoxanthin (for second tertile and for highest tertile compared to lowest tertile, in all cases), moderate intake of matairesonal (for second tertile compared to lowest tertile, in all cases), and high intake of secoisolariciresinol (for highest tertile compared to lowest tertile, in all cases) was associated with poorer survival.

**Table 3:** Associations between intake of fat-soluble antioxidants and survival following glioma diagnosis^†^, stratified by histology group

Nutrient (unit) Tertiles				All or self-report (SR) only	Grade II		Grade III		Grade IV	
					(143 cases, 72 deaths)		(130 cases, 103 deaths)		(475 cases, 473 deaths)	
					
	Lower limit		Upper limit		HR	95% CI	HR	95% CI	HR	95% CI
Vitamin E (mg)										
T1:	0.8	to	6.4	ALL	1		1		1	
T2:	6.5	to	9.4		1.00	(0.44-2.27)	1.02	(0.60-1.74)	1.15	(0.89-1.50)
T3:	9.5	to	42.1		1.05	(0.33-3.33)	0.37*	(0.17-0.81)	0.81	(0.58-1.12)
T1:	0.8	to	6.4	SR	1		1		1	
T2:	6.5	to	9.4		0.87	(0.38-2.01)	1.75	(0.83-3.69)	1.06	(0.74-1.53)
T3:	9.5	to	42.1		0.82	(0.23-2.95)	1.06	(0.38-2.98)	0.97	(0.62-1.51)
Alpha-carotene (mcg)										
T1:	7.4	to	427.1	ALL	1		1		1	
T2:	428.6	to	916.1		0.64	(0.29-1.40)	1.44	(0.81-2.56)	0.99	(0.76-1.28)
T3:	917.8	to	17008.2		1.10	(0.55-2.22)	0.91	(0.48-1.72)	1.06	(0.80-1.40)
T1:	7.4	to	427.1	SR	1		1		1	
T2:	428.6	to	916.1		0.65	(0.28-1.53)	.12*	(1.08-4.18)	0.88	(0.60-1.27)
T3:	917.8	to	17008.2		1.20	(0.57-2.53)	1.18	(0.57-2.45)	1.18	(0.81-1.72)
Beta-carotene (mcg)										
T1:	257.7	to	2070.1	ALL	1		1		1	
T2:	2070.2	to	3754.1		0.86	(0.43-1.74)	0.95	(0.54-1.68)	1.08	(0.84-1.39)
T3:	3757.6	to	39807.1		0.92	(0.45-1.90)	0.76	(0.37-1.54)	1.11	(0.85-1.46)
T1:	257.7	to	2070.1	SR	1		1		1	
T2:	2070.2	to	3754.1		0.79	(0.36-1.71)	1.36	(0.64-2.90)	1.06	(0.74-1.53)
T3:	3757.6	to	39807.1		1.08	(0.53-2.21)	1.45	(0.61-3.48)	0.97	(0.66-1.41)
Carotenoids (mcg)										
T1:	303.0	to	2501.5	ALL	1		1		1	
T2:	2503.5	to	4658.0		0.89	(0.43-1.84)	1.05	(0.60-1.82)	1.11	(0.87-1.43)
T3:	4662.2	to	51458.1		0.91	(0.46-1.82)	0.68	(0.33-1.41)	1.18	(0.89-1.55)
T1:	303.0	to	2501.5	SR	1		1		1	
T2:	2503.5	to	4658.0		0.74	(0.34-1.64)	1.70	(0.84-3.46)	1.13	(0.78-1.63)
T3:	4662.2	to	51458.1		1.02	(0.50-2.06)	1.21	(0.50-2.90)	1.02	(0.69-1.50)
Lutein (mcg)										
T1:	31.3	to	1285.5	ALL	1		1		1	
T2:	1288.6	to	2687.6		0.69	(0.36-1.35)	0.60	(0.35-1.02)	0.96	(0.74-1.23)
T3:	2694.2	to	51290.4		0.56	(0.26-1.18)	1.03	(0.53-2.01)	1.19	(0.92-1.53)
T1:	31.3	to	1285.5	SR	1		1		1	
T2:	1288.6	to	2687.6		0.70	(0.35-1.43)	1.11	(0.55-2.26)	1.06	(0.73-1.53)
T3:	2694.2	to	51290.4		0.65	(0.30-1.41)	1.86	(0.72-4.78)	1.07	(0.74-1.53)
Lycopene (mcg)										
T1:	0.0	to	914.8	ALL	1		1		1	
T2:	915.8	to	2118.3		1.78	(0.92-3.46)	1.33	(0.77-2.31)	1.27	(0.99-1.62)
T3:	2121.5	to	26788.8		0.81	(0.37-1.80)	0.70	(0.36-1.36)	1.10	(0.85-1.44)
T1:	0.0	to	914.8	SR	1		1		1	
T2:	915.8	to	2118.3		2.31*	(1.12-4.75)	2.17*	(1.02-4.62)	1.10	(0.77-1.57)
T3:	2121.5	to	26788.8		1.00	(0.41-2.44)	2.31	(0.98-5.43)	1.00	(0.69-1.44)
Cryptoxanthin (mcg)										
T1:	0.0	to	79.8	ALL	1		1		1	
T2:	80.2	to	180.3		1.52	(0.70-3.29)	0.66	(0.37-1.18)	1.34*	(1.04-1.73)
T3:	180.4	to	1437.2		0.92	(0.39-2.17)	1.24	(0.66-2.33)	1.57*	(1.20-2.06)
T1:	0.0	to	79.8	SR	1		1		1	
T2:	80.2	to	180.3		1.83	(0.86-3.92)	1.04	(0.51-2.14)	1.19	(0.82-1.72)
T3:	180.4	to	1437.2		0.85	(0.35-2.08)	1.14	(0.52-2.51)	1.32	(0.90-1.93)
Coumestrol (mcg)										
T1:	3.0	to	83.4	ALL	1		1		1	
T2:	84.1	to	145.3		0.99	(0.51-1.90)	0.70	(0.38-1.30)	1.13	(0.88-1.45)
T3:	145.5	to	1315.3		0.77	(0.33-1.75)	1.06	(0.60-1.87)	1.16	(0.88-1.54)
T1:	3.0	to	83.4	SR	1		1		1	
T2:	84.1	to	145.3		1.11	(0.56-2.21)	1.41	(0.67-2.96)	0.98	(0.68-1.40)
T3:	145.5	to	1315.3		0.73	(0.29-1.82)	0.96	(0.45-2.05)	1.06	(0.72-1.57)
Matairesinol (mcg)										
T1:	0.1	to	17.6	ALL	1		1		1	
T2:	17.7	to	34.5		1.17	(0.55-2.49)	0.88	(0.51-1.52)	1.38*	(1.08-1.77)
T3:	34.6	to	218.6		0.78	(0.36-1.69)	0.86	(0.48-1.54)	1.20	(0.92-1.57)
T1:	0.1	to	17.6	SR	1		1		1	
T2:	17.7	to	34.5		1.14	(0.52-2.50)	1.27	(0.59-2.73)	1.34	(0.95-1.89)
T3:	34.6	to	218.6		0.88	(0.38-2.04)	1.00	(0.48-2.09)	1.07	(0.74-1.55)
Secoisolariciresinol (mcg)										
T1:	6.3	to	87.3	ALL	1		1		1	
T2:	87.4	to	146.0		1.25	(0.60-2.61)	0.54*	(0.31-0.93)	1.04	(0.81-1.33)
T3:	146.1	to	698.4		1.95	(0.93-4.10)	0.48*	(0.25-0.92)	1.32*	(1.02-1.72)
T1:	6.3	to	87.3	SR	1		1		1	
T2:	87.4	to	146.0		1.19	(0.52-2.73)	0.75	(0.38-1.49)	0.83	(0.58-1.21)
T3:	146.1	to	698.4		1.41	(0.63-3.15)	0.51	(0.22-1.19)	0.95	(0.66-1.36)
Linoleic acid (g)										
T1:	1.6	to	8.9	ALL	1		1		1	
T2:	9.0	to	14.6		1.21	(0.57-2.58)	1.15	(0.66-2.02)	1.03	(0.80-1.32)
T3:	14.7	to	57.7		0.95	(0.35-2.60)	0.50	(0.25-1.01)	0.87	(0.63-1.19)
T1:	1.6	to	8.9	SR	1		1		1	
T2:	9.0	to	14.6		1.14	(0.51-2.53)	0.88	(0.41-1.89)	0.85	(0.60-1.21)
T3:	14.7	to	57.7		1.17	(0.41-3.35)	0.59	(0.23-1.52)	0.96	(0.60-1.54)

^† ^adjusted for reporting status (all cases vs self reported cases only), age at diagnosis (<48,48-65,>65), treatment (radiation, chemo, surgery - yes/no), education (<12, 12, >12), marital status (yes/no), total calories (continuous), pack years (median = 18.5), and age at first alcoholic drink (<18,18-20,>20).

* p < 0.05

Table 4 presents hazard ratios for tertiles of daily intake of water-soluble antioxidants adjusted for the same set of covariates as the models shown in Table 3. Among Grade II patients, moderate intake of folate was associated with enhanced survival (HR = 0.45, 95% CI = 0.20 to 0.99, for second tertile compared to lowest tertile, in all cases). Moderate intake of formonoetin among Grade III patients was also associated with enhanced survival. Among Grade IV patients, high intake of vitamin C and genistein as well as moderate intake of formonoetin, in all cases, was associated with poorer survival.

**Table 4:** Associations between intake of water-soluble antioxidants and survival following glioma diagnosis^†^, stratified by histology group

Nutrient (unit) Tertiles				All or self-report (SR) only	Grade II		Grade III		Grade IV	
					(143 cases, 72 deaths)		(130 cases, 103 deaths)		(475 cases, 473 deaths)	
					
	Lower limit		Upper limit		HR	95% CI	HR	95% CI	HR	95% CI
Vitamin C (mg)										
T1:	4.5	to	84.1	ALL	1		1		1	
T2:	84.1	to	149.6		1.33	(0.72-2.48)	0.71	(0.40-1.27)	1.23	(0.96-1.57)
T3:	149.6	to	869.6		0.52	(0.23-1.20)	1.07	(0.56-2.03)	.44*	(1.09-1.91)
T1:	4.5	to	84.1	SR	1		1		1	
T2:	84.1	to	149.6		1.21	(0.63-2.34)	1.04	(0.50-2.16)	1.2	(0.84-1.72)
T3:	149.6	to	869.6		0.56	(0.21-1.47)	1.18	(0.51-2.75)	1.28	(0.87-1.90)
Formononetin (mcg)										
T1:	0.1	to	9.3	ALL	1		1		1	
T2:	9.4	to	23.0		1.08	(0.55-2.15)	47*	(0.25-0.89)	.27*	(1.00-1.61)
T3:	23.1	to	155.6		1.08	(0.46-2.52)	0.79	(0.43-1.43)	1.04	(0.79-1.37)
T1:	0.1	to	9.3	SR	1		1		1	
T2:	9.4	to	23.0		1.18	(0.57-2.42)	0.67	(0.29-1.53)	1.16	(0.82-1.63)
T3:	23.1	to	155.6		0.96	(0.41-2.29)	0.56	(0.27-1.14)	1.03	(0.71-1.49)
Genistein (mcg)										
T1:	8.1	to	141.3	ALL	1		1		1	
T2:	141.7	to	291.3		1.18	(0.57-2.43)	1.18	(0.66-2.12)	1.2	(0.92-1.56)
T3:	291.6	to	44265.2		1.05	(0.40-2.74)	1.25	(0.69-2.27)	1.35*	(1.00-1.81)
T1:	8.1	to	141.3	SR	1		1		1	
T2:	141.7	to	291.3		0.93	(0.42-2.07)	1.53	(0.71-3.29)	1.02	(0.70-1.48)
T3:	291.6	to	44265.2		0.78	(0.29-2.15)	1.56	(0.65-3.74)	1.42	(0.96-2.10)
Daidzein (mcg)										
T1:	10.1	to	269.0	ALL	1		1		1	
T2:	270.1	to	440.6		1.77	(0.96-3.26)	0.71	(0.40-1.28)	1.06	(0.83-1.36)
T3:	440.6	to	36886.6		1.7	(0.70-4.14)	1.01	(0.55-1.85)	1.13	(0.86-1.49)
T1:	10.1	to	269.0	SR	1		1		1	
T2:	270.1	to	440.6		1.54	(0.75-3.17)	1.05	(0.49-2.25)	1.2	(0.84-1.72)
T3:	440.6	to	36886.6		1.21	(0.46-3.20)	0.7	(0.29-1.66)	1.08	(0.75-1.57)
Biochanin A (mcg)										
T1:	0.0	to	15.4	ALL	1		1		1	
T2:	15.4	to	37.8		1.03	(0.53-2.01)	0.84	(0.47-1.51)	1.27	(0.99-1.62)
T3:	37.8	to	334.7		0.6	(0.28-1.30)	0.91	(0.45-1.88)	1.26	(0.97-1.64)
T1:	0.0	to	15.4	SR	1		1		1	
T2:	15.4	to	37.8		1.24	(0.62-2.49)	0.56	(0.26-1.20)	0.92	(0.64-1.33)
T3:	37.8	to	334.7		0.56	(0.24-1.32)	0.66	(0.27-1.57)	1.02	(0.70-1.50)
Calcium (mg)										
T1:	85.0	to	499.9	ALL	1		1		1	
T2:	501.6	to	807.7		1.23	(0.61-2.47)	0.7	(0.39-1.24)	1.06	(0.82-1.37)
T3:	808.1	to	2686.8		0.95	(0.35-2.53)	1.22	(0.59-2.55)	0.87	(0.64-1.19)
T1:	85.0	to	499.9	SR	1		1		1	
T2:	501.6	to	807.7		1.5	(0.70-3.24)	1.32	(0.65-2.67)	1.06	(0.74-1.52)
T3:	808.1	to	2686.8		1.56	(0.58-4.15)	2.27	(0.93-5.53)	0.81	(0.53-1.24)
Folate (mcg)										
T1:	56.5	to	297.0	ALL	1		1		1	
T2:	297.4	to	434.7		0.45*	(0.20-0.99)	1.05	(0.62-1.78)	0.84	(0.65-1.09)
T3:	435.1	to	1530.0		0.35	(0.12-1.01)	0.59	(0.28-1.24)	1.08	(0.78-1.50)
T1:	56.5	to	297.0	SR	1		1		1	
T2:	297.4	to	434.7		0.49	(0.22-1.12)	1.29	(0.61-2.73)	0.82	(0.57-1.17)
T3:	435.1	to	1530.0		0.41	(0.13-1.29)	2.26	(0.77-6.60)	1.09	(0.68-1.74)

^† ^adjusted for reporting status (all cases vs self reported cases only), age at diagnosis (<48,48-65,>65), treatment (radiation, chemo, surgery - yes/no), education (<12, 12, >12), marital status (yes/no), total calories (continuous), pack years (median=18.5), and age at first alcoholic drink (<18,18 - 20,>20).

* p < 0.05

Hazard ratios for tertiles of the Antioxidant Index and the macronutrients are presented in Table 5.

**Table 5:** Associations of total antioxidant index and macronutrients with glioma survival^†^, stratified by histology group

Nutrient (unit) Tertiles				All or self-report (SR) only	Grade II		Grade III		Grade IV	
	Lower limit		Upper limit		(143 cases, 72 deaths)		(130 cases, 103 deaths)		(475 cases, 473 deaths)	
					
					HR	95% CI	HR	95% CI	HR	95% CI
Antioxidant index										
T1:	50.1	to	1961.9	ALL	1		1		1	
T2:	1963.2	to	3263.0		1.34	(0.70-2.54)	1.31	(0.78-2.18)	1.02	(0.78-1.32)
T3:	3266.1	to	13512.6		0.67	(0.30-1.47)	1.04	(0.58-1.88)	0.36*	(1.02-1.81)
T1:	50.1	to	1961.9	SR	1		1		1	
T2:	1963.2	to	3263.0		1.41	(0.71-2.80)	1.36	(0.62-2.94)	1.18	(0.82-1.68)
T3:	3266.1	to	13512.6		0.74	(0.31-1.77)	1.03	(0.49-2.16)	1.07	(0.72-1.59)
Total Protein (g)										
T1:	11.4	to	49.5	ALL	1		1		1	
T2:	49.6	to	70.8		0.44*	(0.20-0.95)	1.44	(0.75-2.74)	1.08	(0.83-1.40)
T3:	71.1	to	241.5		0.32	(0.10-1.04)	1.73	(0.78-3.83)	0.64*	(0.43-0.95)
T1:	11.4	to	49.5	SR	1		1		1	
T2:	49.6	to	70.8		0.40*	(0.18-0.93)	1.57	(0.74-3.31)	1.13	(0.78-1.62)
T3:	71.1	to	241.5		0.53	(0.15-1.81)	1.18	(0.42-3.27)	0.88	(0.52-1.50)
Total Fat (g)										
T1:	10.2	to	47.1	ALL	1		1		1	
T2:	47.2	to	72.7		1.33	(0.60-2.96)	0.77	(0.43-1.38)	1.13	(0.87-1.47)
T3:	72.9	to	213.0		0.88	(0.29-2.66)	0.73	(0.35-1.51)	1.10	(0.74-1.63)
T1:	10.2	to	47.1	SR	1		1		1	
T2:	47.2	to	72.7		1.10	(0.47-2.54)	0.67	(0.29-1.53)	1.06	(0.73-1.53)
T3:	72.9	to	213.0		0.81	(0.26-2.53)	0.74	(0.30-1.85)	1.11	(0.61-2.01)
Total Cholesterol (mg)										
T1:	17.2	to	166.1	ALL	1		1		1	
T2:	166.2	to	272.7		0.53	(0.24-1.14)	0.86	(0.49-1.49)	1.50*	(1.17-1.93)
T3:	273.0	to	1322.0		0.76	(0.31-1.86)	0.49*	(0.25-0.95)	1.31	(0.97-1.78)
T1:	17.2	to	166.1	SR	1		1		1	
T2:	166.2	to	272.7		0.59	(0.25-1.41)	1.04	(0.53-2.03)	1.39	(0.98-1.97)
T3:	273.0	to	1322.0		1.00	(0.38-2.59)	0.68	(0.28-1.63)	1.13	(0.71-1.80)

^† ^adjusted for reporting status (all cases vs self reported cases only), age at diagnosis (<48,48-65,>65), treatment (radiation, chemo, surgery - yes/no), education (<12, 12, >12), marital status (yes/no), total calories (continuous), pack years (median = 18.5), and age at first alcoholic drink (<18,18-20,>20).

* p < 0.05

Moderate intake of total protein (HR = 0.44, 95% CI = 0.20 to 0.95, for second tertile compared to lowest tertile, in self-reported cases only; HR = 0.40, 95% CI = 0.18 to 0.93, for second tertile compared to lowest tertile, in all cases) among Grade II patients was associated with enhanced survival; a similar result was observed among Grade IV patients with high intake of total protein. Among Grade III patients, high intake of total cholesterol, in all cases, was associated with better survival. Moderate intake of total cholesterol, in all cases, was associated with poorer survival among Grade IV patients. The only statistically significant results for Antioxidant Index were observed in Grade IV patients where the highest tertile of intake, in all cases, (HR = 1.36, 95% CI = 1.02 to 1.81) was associated with poorer survival.

## Discussion

Intake of dietary antioxidants and other nutrients may be part of the risk profile associated with enhanced or diminished survival following a diagnosis of glioma. Our study results offer some insights among glioma patients diagnosed with Grade II and Grade III tumors, and possibly Grade IV tumors. Our analysis included a large glioma patient cohort recruited over a 9-year interval when treatment practices remained generally unchanged. Our study cohort is comparable to other cohorts of glioma patients in the distribution of patient and clinical characteristics such as age at diagnosis and pattern of treatment.

Although most individual antioxidant effect estimates in our analyses were not statistically significant, generally the magnitude and direction of the hazard ratios in all three histology groups was consistent across self-reported cases and all cases. Some inconsistent results, however, were observed where moderate intake of lycopene was associated with poorer survival among the self-reported cases only in both Grade II and Grade III patients. A similar result for moderate intake of alpha-carotene was observed in Grade III patients. Among these two carotenoids, lycopene has been shown to inhibit progression of prostate, breast and lung cancer cell lines [26]. However while carotenoids in general exhibit antioxidant behavior at low oxygen partial pressures (below 150 Torr), they can lose antioxidant potential and become pro-oxidant at higher pressures of oxygen [26].

Significantly enhanced survival estimates were observed for moderate intake of folate as well as total protein intake among Grade II patients; this was consistent across self-reported cases and all cases. Among Grade III patients, significantly enhanced survival occurred with high intake of vitamin E, moderate intake of formononetin, and both moderate and high intake of secoisolariciresinol in all cases. Laboratory analysis has shown that vitamin E (particularly α-tocopheryl succinate and tocopherols) inhibits the angiogenic factor, vascular endothelial growth factor (VEGF), which is induced by glioma tumors, thus having the potential to limit glioma cell proliferation [55]. Gamma-tocopherols have also been shown to decrease PKC upstream and retinoblastoma(Rb) phosphorylation downstream controlling cell cycle production in C-6 glioma cells [56]. Tedeschi-Blok et al [46] observed a chemopreventive effect for moderate intake of vitamin E and formononetin, and high intake of secoisolariciresinol in a comparison of all (not stratified by histology group) glioma cases (same cohort as current study) vs controls.

Among Grade IV patients the magnitude of the hazard ratios was generally consistent across self-reported cases and all cases. Among all Grade IV cases, a number of antioxidants and other nutrients were significantly associated with poorer survival. These included high intake of the total Antioxidant Index, vitamin C, genistein, and secoisolariciresinol; modertate intake of formononetin, matairesinol and total cholesterol; moderate and high intake of cryptoxanthin. Moderate intake of total protein was associated with significantly enhanced survival. That significant results were not observed in the self-reported cases only may indicate systematic misclassification errors attributable to proxy respondents who were not entirely accurate in recalling the case's dietary habits, or to cases that were too ill to recall their actual eating patterns with clarity. The hazard ratios for secoisolariciresinol and formononetin in the Grade IV group are in the opposite direction from the corresponding hazard ratios estimated among Grade II and Grade II patients. While such difference could be due to chance, it does highlight the need for stratifying data analyses by histology group, which is a strength of the current study. Moreover, histology Grades III and IV have shorter survival following diagnosis of glioma than Grade II; and Grade IV, which has almost 100% mortality within 5 years of diagnosis, is also distinguished from Grade III. Therefore, the diet prior to diagnosis is closer to time of death for higher than lower Grade glioma patients.

The difference in the associations between antioxidants and survival of patients with different glioma grades may be explained by the specific tumor biology, differences in the interaction between the treatment and antioxidants, or by chance. There are several indications that the detected associations can not be explained by chance alone. Where the same antioxidant\nutrient was significant in more than one grade, the hazard ratios were generally in the same direction in several instances: one example is the association with lycopene among Grade II and Grade III patients; another example is total protein in Grade II and Grade IV patients.

Statistically significant results were not confined to one particular subclass of antioxidants, but were found among isoflavones (e.g. genistein), carotenoids (e.g. lycopene), lignans (e.g. matairesinol), vitamin C, vitamin E and folate. Our study also observed significant results among both fat- and water- soluble antioxidants.

Our study used patients' responses to a diet questionnaire as the primary data source for converting this information into grams of specific antioxidant consumed per day by applying an appropriate algorithm. We recognize that internal exposure dose (usually measured as plasma concentration) to antioxidants may be confounded by individual difference in absorption. However, at least for carotinoids, a correlation has been demonstrated between questionnaire-derived intake and plasma levels [57,58].

Recent developments in treatment of malignant gliomas have provided some extension of survival time through the concomitant administration of temozolomide (TMZ) chemotherapy following surgery and radiotherapy [59]. Since our study cohort preceded the widespread introduction of TMZ as standard of care for glioblastoma (grade IV) in 2005, the enhanced survival associated with TMZ use is not reflected in this patient cohort. However a strength of our study is that the association between dietary antioxidants and glioma patient survival is not confounded by patients receiving TMZ.

Given the large number of associations examined, we acknowledge that some findings may be merely due to chance. Although there remains controversy regarding the use of some adjustment for multiple associations or comparisons in observational studies (e.g. Bonferroni), it is our position not to use them. We base our decision in part on the rationale outlined by Rothman [60] and Savitz [61]. Rothman asserts that the adjustment, while decreasing type I error, would increase the risk of type II error for associations that are not null. He furthermore states that "a policy of not making adjustment for multiple comparisons is preferable because it will lead to fewer errors of interpretation when the data under evaluation are not random numbers but actual observations on nature". As recommended, we have made clear the number and nature of statistical tests, allowing audiences their own interpretation of study findings.

## Conclusions

The associations observed in our study suggest that the influence of some non-enzymatic antioxidants on survival following a diagnosis of malignant glioma are inconsistent and vary by histology group. In the same cohort of glioma patients, a case-control study found a number of significant inverse associations between antioxidant intake and gliomagenesis [46]; however our current study of antioxidant intake and glioma survival did not observe the same number of inverse associations. Further research in a large sample of glioma patients is needed to confirm or refute our study results.

## Competing interests

The authors declare that they have no competing interests.

## Authors' contributions

GD planned, implemented and interpreted results from this study's data/survival analyses, and was principal author of this manuscript. LM and TR, project coordinators, prepared study data sets, conducted some of the statistical analyses, and performed manuscript revision. AT conducted statistical analyses and performed manuscript revision. CQ provided biostatistical expertise in the design and conduct of the statistical analyses and the interpretation of study results, and performed manuscript revision. DI provided expertise in the analysis of dietary data, developed background information for the manuscript, interpreted study results, and performed manuscript revision. MW, overall PI of the San Francisco Bay Area Adult Glioma study, oversaw subject recruitment and data collection, oversaw statistical analyses, interpreted results, and performed manuscript revision. All authors read and approved the final manuscript.

## Pre-publication history

The pre-publication history for this paper can be accessed here:

http://www.biomedcentral.com/1471-2407/10/215/prepub
